# Digital Visual Communication for Public Health: Design Proposal for a Vaccinated Emoji

**DOI:** 10.2196/35786

**Published:** 2022-04-07

**Authors:** Tamara Sonia Boender, Noah Louis-Ferdinand, Gideon Duschek

**Affiliations:** 1 Surveillance Unit Department of Infectious Disease Epidemiology Robert Koch Institute Berlin Germany; 2 Voices for Vaccines Decatur, GA United States; 3 Ommado Amsterdam Area Netherlands

**Keywords:** vaccination, emoji, design, science communication, infodemic management, vaccine confidence, digital communication

## Abstract

In the 21st century, the internet and particularly social media have become essential platforms for the spread of health information (including misinformation and disinformation). One of the distinguishing features of communication on these platforms is the widespread use of emojis. Though seemingly trivial emojis are now used by many if not most public health figures and organizations alongside important health updates. Much of that information has had to do with vaccination. Vaccines are a critical public health tool but one surrounded by falsehoods, phobias, and misinformation fueling vaccine hesitancy. Part of that has to do with their lack of positive representation on social media (eg, the syringe emoji is a plain needle, which for many people is an uncomfortable image). We thus argue that vaccination deserves an entirely new emoji to communicate vaccine confidence and discuss a design proposal for a vaccinated emoji that has gained traction in the global public health community.

## Digital Media and Vaccine Hesitancy

Communicating online has reached an all-time high for both scientists and the public, and has been catalyzed by public health and social measures in the context of the COVID-19 pandemic. The potential value of emojis in the context of medical communication and care has recently come into the limelight [1], given that their use is a defining feature of many of the online platforms. For instance, a recent survey of 68 million tweets found that one in five contained an emoji [2]. These emojis are often used to discuss current events, and indeed in the early phases of the COVID-19 pandemic, there was a clear increase on the *microbe* emoji 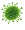 and *face with medical mask* 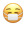 emoji [3]. As the BioNTech/Pfizer and Moderna mRNA vaccines began to roll out, there was then a subsequent increase in the use of the *syringe* emoji 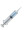 [4]. However, for many people, it did not feel representative since the syringe is meant to represent blood donation; influential medical doctor Jeremy Faust referred to it on Twitter as an “inaccurate bloody syringe” [3,5,6]. Several platforms responded by removing the blood from the *syringe* emoji 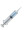. Yet this does not address the fact that for many people *needles themselves* 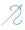 come with a host of negative associations. Needle phobia affects some 25% of adults [7], often causing them to delay or turndown vaccination. Even for those without a phobia, the symbol is still tied to a painful experience. This is despite vaccination being a profoundly protective intervention central to public health. We therefore argue that there is currently no accurate emoji for vaccination.

This is no small problem. In 2019, the World Health Organization (WHO) listed vaccine hesitancy, or the reluctance to vaccinate despite availability, among the top 10 threats to global health [8]. This was before the pandemic, when the lifesaving power of vaccines had come to be taken for granted, despite them being one of the best tools we have to prevent moderate and severe disease. Take for example the human papillomavirus vaccine that has the remarkable ability to prevent cancer. It has all but eliminated cervical cancer in women in the United Kingdom born after the mid-1990s. Yet, the WHO/United Nations Children’s Fund (UNICEF) estimate that average performance coverage for the final dose among member states is only about 54% [9]. Lack of uptake year after year has surely caused a great deal of unnecessary suffering.

Vaccine hesitancy becomes an especially pressing problem in the context of pandemics, such as the 2009-2010 H1N1 (hemagglutinin type 1 and neuraminidase type 1) novel influenza A pandemic and the current COVID-19 pandemic. This is especially costly given that the COVID-19 vaccines offer an extraordinary degree of protection against severe clinical outcomes, including death. In 33 countries across the WHO European Region, an estimated 470,000 lives have been saved among those aged ≥60 years in 2021 alone [10]. Yet, only about one-third of the region was fully vaccinated by October 2021, with hesitancy being a major barrier to uptake [11].

Improving vaccine confidence, whether in Europe or elsewhere, will require more effective messaging. In the social media age [12], this means coming up with new tools for digital communication, supporting online risk communication and community engagement. We would specifically highlight the use of digital symbols to create shifts in attitude toward real-world phenomena. For instance, Plan International ran a campaign back in 2017 to create a “period emoji” [13]. Their stated intent was to reduce stigma and push back against harmful beliefs by coming up with a more modern way to communicate about menstruation. The product (designed alongside National Health Services Blood and Transplant) was the *drop of blood* emoji 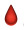. In another case, Apple decided to change its *pistol* emoji to a *water pistol* emoji 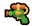 [14,15]. This was widely perceived to be an effort to reduce progun sentiment given that the company also vetoed a rifle emoji.

With regard to public health, the creation of emojis is not a new idea. For example, the *mosquito* emoji 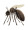 was proposed in 2016 in the context of the Zika virus outbreak [16]. The mosquito is the deadliest animal on earth due to spreading diseases like Zika, malaria, dengue, and yellow fever. The emoji helps health professionals to communicate with the public about the presence of mosquitoes and allows researchers to promote their work around mosquito-borne diseases more easily via social media and other digital platforms. Of note, the original *mosquito* emoji 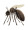 design has been added to the permanent collection of the Victoria and Albert Museum in London [17].

In this viewpoint, we build the case for a *vaccinated* emoji to meet the current public health need for better digital communication on vaccine confidence.

## The Design Proposal: The Vaccinated Emoji

**Figure 1:**
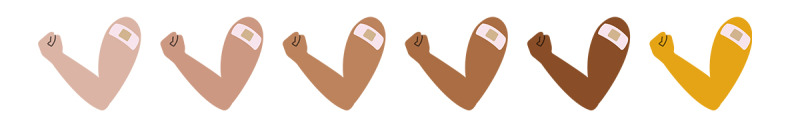
The proposed design of the vaccinated emoji.

We present a design for a new emoji that accurately reflects the benefits of vaccination: the *vaccinated* emoji (Figure 1). Following the request for an alternative to the *syringe* emoji 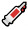 as posted on Twitter by author TSB on January 1, 2021 [18], she contacted author GD for his ideas for the design of a vaccinated emoji that conveys the message of both the act and sentiment of vaccination: the protection of being vaccinated. The design process unites the strengths of health sciences and epidemiology (TSB) with design (GD). As a first step, it made sense to remove the blood from the syringe and leave it clear [3]. However, the emoji would still contain a sharp needle, which for many people is an uncomfortable image. We wanted to create a symbol of hope, which represents protection against once-deadly diseases.

We aimed for a generic arm as a base for the emoji, representing all ages, genders, and skin tones, based on the *flexed biceps* emoji 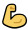. The *flexed biceps* emoji 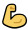 is a top-ranked emoji in its class (body parts) and often used to indicate strength, success, overcoming a struggle, or rolling up your sleeve to receive a vaccine [19]. We then added the *adhesive bandage* emoji 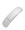 over the deltoid to couple this symbol of strength with one of protection and care. Their combination enables the user to communicate the benefits of vaccination in an intuitive, playful way. The initial design of the *vaccinated* emoji by author GD, introducing the relatable and protective image of an arm with a bandage, was posted on Twitter by January 6, 2021 [20]. Of note, accurate placement of the bandage on the deltoid muscle has been challenging, and the bandage itself is also disproportionally large. However, disproportionate scale in the relationship between parts is typical for emojis. For example, the *crying face* emoji 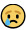 has a relatively large tear because it would not be visible otherwise. The emoji was designed using the graphics editor and design programs Photoshop (Adobe Inc) and Illustrator (Adobe Inc).

To enable universal and international access to the *vaccinated* emoji, TSB and GD have submitted the design proposal to the Unicode Consortium—who formally manages emoji approval and implementation across devices, platforms, and languages—in 2021. The Unicode Standard can ensure global access to the emojis across all platforms through the associated license agreement [21,22]:

Submitter hereby acknowledges and agrees that they are submitting the Proposed Emoji for inclusion in the Standards, and further acknowledges and agrees that, if included in the Standards, the Proposed Emoji will be freely licensed by Unicode to all users around the world under the Unicode Licenses.

The Unicode Emoji Subcommittee only selects a limited number of emojis that can be encoded and unfortunately did not select the vaccinated emoji proposal to move forward in 2021. Nonetheless, we are currently revising the emoji proposal for resubmission in 2022. It is not uncommon for emojis to be proposed multiple times before successful implementation, which was also the case for the *mosquito* emoji 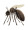 [23]. To ensure optimal advocacy for the vaccinated emoji, we also reached out to Emojination, a grassroots organization that has previously been successful at advocating for inclusive and representative emojis, including the *hijab* (person headscarf) emoji 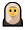, as well as the infectious disease prevention–related emojis for *soap* 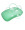 and *microbe* 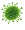 [24].

To increase uptake where vaccines are available and affordable, thoughts, feelings, and social processes can motivate getting vaccinated [25]. People are more likely to get vaccinated if those in their social circle do so as well [26]. Sending an emoji to friends and family or adding one to a tweet is literally a matter of two clicks or phone taps. The vaccinated emoji is a low-key way of both seeing and signaling others about their support of vaccination and sharing the news that they just got vaccinated. The vaccinated emoji pairs well with ongoing trends such as the vaccine selfie, where social media users post photos of themselves at a clinic with a bandage over their arm [27]. Perhaps most importantly, the emoji can be used alongside personal stories, posts, and chats or group chats about vaccination to mark the event and reinforce one’s reasons for getting vaccinated. This is important both because antivaccine messages are much more likely to make use of emotionally compelling stories [28] and users are more likely to remember and be deterred by stories of harm [29].

## Communicating Vaccine Confidence

The vaccine emoji was created by TSB, a health scientist and field epidemiologist, and GD, a designer. After posting the design proposal to Twitter on January 1, 2021 [18], it quickly gained 40,000 impressions (user views). This prompted NRC—a Dutch newspaper with 4.1 million monthly readers—to pick up and report on the trend the following week [30]. The emoji has been encouraged by campaigns such as Voices for Vaccines *#WhyIVax* on Twitter where trusted doctors and public health professionals gave positive examples, often with a photo of a loved one they were protecting through vaccination. The idea behind the campaign—which author NLF played a role in—was to give other users the courage to speak up about vaccines. The powerful flex of the vaccine emoji conveys confidence in one’s convictions and helps animate the important yet dry facts that comprise many provaccine posts [28]. People are more likely to engage in social media trends that take minimal effort. The intuitive nature of the action—though itself small—can have compounding effects due to the nature of virality and the fact that vaccination in one season predicts vaccination in the following seasons [26]. An emoji is the most organic, least heavy-handed way of getting that point across.

Later, NLF, who was a social media specialist for the Task Force for Global Health, began to help popularize the emoji. We reached out to a number of people through Twitter and rallied the global and public health community for *#WorldEmojiDay*, July 17 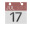. International attention skyrocketed, and within a day, the proposal had been endorsed by many, including senior leaders at the WHO including Dr Tedros Adhanom Ghebreyesus (Director-General) and Dr Maria van Kerkhove (COVID-19 technical lead) [31,32]. The Task Force for Global Health metrics show that the emoji had received 300,000 impressions on their page alone in a matter of 3 days, and one can see many tweets from ordinary users endorsing the emoji and posting selfies with flexed arms. Much of this support seemed to come from the fact that the emoji is an explicitly positive symbol. Hashtags, which can also be used to rally support for a cause, have in the past year often been a site of misinformation as they can be “hijacked” easily. They are also less intuitive with respect to what they mean or how they are to be used. The success of the *vaccinated* emoji is driven by its accessibility, ease of use, and universal appeal, winning praise from commentators as diverse as Heidi Murkoff (the author of the What to Expect When You’re Expecting series of pregnancy guides) and Toyin Saraki (global health advocate, health care philanthropist, and the Founder-President of Wellbeing Foundation Africa). The emoji going viral on Twitter spilled over to media coverage in the United States [6] and the Netherlands [33]. We see this kind of organic support as crucial to the success of any public health symbol. We also see scientific communication as an underleveraged component of public health. This means we should follow organic interest to create public investment in health campaigns. The vaccine emoji does precisely that.

Since World Emoji Day 2021, the *vaccinated* emoji has become a fixture in the space of digital health advocacy. By August 2021, the emoji was selected for presentation at the Stanford Infodemic Conference on Social Media and COVID-19 Misinformation [34]. In October 2021, GD was commissioned by the German Federal Government to design an icon for their national contact-tracing app (Corona-Warn-App, Version 2.13 [35]) to visually strengthen the call to get vaccinated, drawing heavily upon the vaccine emoji. Both events speak to a growing recognition that the public health community must adopt symbols that meet the public where they are at. Though still considered trivial by some, our need to shift tactics is more than urgent in a time when vaccine hesitancy has hardened into refusal by some and a sense of defeat by the rest. Now more than ever, an intuitive participatory approach to health communications is needed to support vaccine confidence. At times of physical distancing and increased use of digital communications, such as app groups and social media, emojis add to the sentiment of conversation around vaccination. Furthermore, the visual language of the *vaccinated* emoji (ie, showing an arm with a bandage) has been integrated into health promotion materials by national and international public health institutes with large reach, such as the Robert Koch Institute (Germany) [36], the US Centers for Disease Control and Prevention [37,38], and European Centre for Disease Prevention and Control [39], as well as the Google Doodle with the message “Get vaccinated. Wear a mask. Save lives” [40].

One year after its initial design, the proposal to add an emoji for vaccination gained international support from public health and medical professionals, national and international nongovernmental organizations, the infodemic management community, and the public. We endorse previous advocacy for expanding the available set of medical emojis and wish to amplify the need for emojis for public health by our proposed vaccinated emoji (Figure 1). Big tech companies have taken the first steps to provide appropriate visual digital communication tools for public health on the topic of vaccination, such as the *Vaccines for All* WhatsApp Sticker Pack in collaboration with the WHO [41] and the Twitter hashflag for the hashtag *#vaccinated* 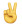 [42]. Furthermore, UNICEF is running a major social media campaign using the hashtag #vaccinated to help spread the word that COVID-19 vaccines are safe and effective by encouraging people to post pictures of their bandage [43]. These major social media campaigns highlight the need for a universal icon supporting this hashtag (potentially also used as a hashflag). The utility of a symbol cannot be divorced from its popularity, and from a communications perspective, the latter is a primary determinant of value.

Emojis are here to stay in our societies and have played a role not only in the fields of computer science and communication but also in marketing, behavioral science, linguistics, psychology, medicine, and education [44,45]. Importantly, social development is linked to emojis, such as the availability for emojis with different skin tones and genders, menstruation, and pregnancy. Encoding the vaccine emoji in the global Unicode Standard would help to make this symbol available for direct use for all to facilitate swift adaptation to ongoing (online) narratives regarding vaccination, which is much needed in the context of the pandemic and infodemic. Ultimately, the vaccinated emoji aims to be more than just an icon: a tool for public health.

## Abbreviations

H1N1: hemagglutinin type 1 and neuraminidase type 1
UNICEF: United Nations Children's Fund
WHO: World Health Organization

## Disclaimer

All rights of the *vaccinated* emoji (Figure 1 in this work) are preserved to the author GD. For the illustrative purpose of publication for this article, JMIR is granted permission to use this image (Figure 1) with open access. The *vaccinated* emoji design proposal will be resubmitted to Unicode in 2022, in agreement with the Emoji Proposal Agreement & License. In this legal agreement, we warrant that the proposed emoji is available for free and open licensing, and grant to the Consortium broad rights, specifically a nonexclusive, irrevocable, perpetual, worldwide, royalty-free license to encode the proposed emoji and to sublicense it under the Consortium’s various open-source licenses.
